# Do different factors influence whether girls versus boys meet ADHD diagnostic criteria? Sex differences among children with high ADHD symptoms

**DOI:** 10.1016/j.psychres.2018.12.128

**Published:** 2019-02

**Authors:** Florence Mowlem, Jessica Agnew-Blais, Eric Taylor, Philip Asherson

**Affiliations:** a Social, Genetic, and Developmental Psychiatry Centre (SGDP), Institute of Psychiatry, Psychology & Neuroscience, King's College London, DeCrespigny Park, Denmark Hill, London SE5 8AF, UK; b Department of Child and Adolescent Psychiatry, Institute of Psychiatry, Psychology & Neuroscience, King's College London, London, UK

**Keywords:** Attention-deficit/hyperactivity disorder, Gender differences, Diagnosis, Non-referred/population-based

## Abstract

We investigate if different factors influence whether girls versus boys meet diagnostic criteria for attention-deficit/hyperactivity disorder(ADHD) among children with high ADHD symptoms. Participants were 283 children aged 7–12 from a population-based study. Girls and boys meeting diagnostic criteria for ADHD, based on an objective investigator-based interview, were compared to children who did not meet criteria despite high symptoms on a rating-scale measure of ADHD. We assessed factors that could differentially relate to diagnosis across girls and boys including ADHD symptoms, co-occurring behavioural/emotional problems and impairment, and sex-effects in rater perceptions of ADHD symptoms. While overall similar factors distinguished girls and boys who met diagnostic criteria from high-symptom peers, effect sizes were larger in girls. Emotional problems were particularly salient to distinguishing diagnosed versus high-symptom girls but not boys. Parents rated boys meeting diagnostic criteria as more impaired than high-symptom boys but did not do so for girls, and under-rated diagnosed girls’ hyperactive/impulsive symptoms compared to more objective interview assessment, with the opposite observed in boys. Results suggest girls’ ADHD may need to be made more prominent by additional behavioural/emotional problems for them to meet full diagnostic criteria and that sex differences in parental perceptions of ADHD behaviours and impairment exist.

## Highlights

• Emotional problems were particularly salient to distinguishing diagnosed vs high-symptom girls.
• Parents rated diagnosed boys as more impaired than high-symptom boys, but not do so in girls.
• Parents underrated diagnosed girls’ hyperactivity/impulsivity compared to objective interview.
• Parents overrated diagnosed boys’ hyperactivity/impulsivity compared to objective interview.

## 1 Introduction

A well-established feature of attention-deficit/hyperactivity disorder (ADHD) is the large sex difference in referral and diagnostic rates. The ratio of boys to girls diagnosed with ADHD in childhood falls in the range of 2:1 to 10:1 (Arnett et al., 2015, Biederman et al., 2002, Novik et al., 2006, Ramtekkar et al., 2010, Willcutt, 2012), with higher ratios seen in clinical compared to population samples. This difference highlights the possibility that ADHD may be underdiagnosed in girls in clinical practice (Ramtekkar et al., 2010). Further, it suggests that investigating sex differences in population-based samples could extend and enrich our understanding of the ADHD construct beyond that of clinical samples.

A common explanation for the observed sex differences in referral and diagnosis is that girls with ADHD are more likely to present with predominantly inattentive symptoms, rather than the more potentially disruptive hyperactive/impulsive symptoms, as well as greater levels of internalising symptoms such as anxiety and depression which might lead to alternative diagnoses (Arnold, 1996, Quinn, 2008). In contrast, boys with ADHD are often characterised as presenting with more hyperactivity/impulsivity, and co-occurring behavioural problems such as oppositional defiant and conduct disorder (Arnold, 1996, Quinn, 2008). It has also been shown that proportionally more boys than girls with ADHD annoy or upset their teachers, and that parents see the ‘feminine’ ADHD diagnostic items as less problematic than the ‘masculine’ ones (Graetz et al., 2005, Ohan and Johnston, 2005). It is highly likely that these explanations, along with the greater rate of diagnosis in boys, has led to an ADHD stereotype of a ‘disruptive boy’, which may influence how behaviour in boys and girls is perceived by individuals key to the referral and diagnostic process (e.g., parents and teachers). Consistent with this view, it has been shown that parents perceived the DSM-IV ADHD criteria as being descriptive of boys (Ohan and Johnston, 2005).

If sex-specific stereotypes of ADHD exist, then it is possible parents and teachers may not as readily recognise manifestations of ADHD in girls compared to boys. Furthermore, sex differences in recognition of ADHD may in part reflect bias in the diagnostic criteria, or the way they are applied. For example, if diagnostic criteria are based on a male presentation of the disorder then females may be less likely to meet full diagnostic criteria (Hong et al., 2014).

The male preponderance could also be due to underlying aetiology of ADHD in relation to a ‘female protective effect’. This model proposes that females require greater genetic and environmental ‘load’ or exposure to factors associated with ADHD to manifest the same degree of impairment and warrant diagnosis as males with ADHD (Eriksson et al., 2016, Rhee and Waldman, 2004, Taylor et al., 2016). Partial support for this hypothesis has been demonstrated, for example siblings of females with ADHD have been shown to display greater familial risk of ADHD compared to siblings of males with ADHD (Martin et al., 2018, Rhee and Waldman, 2004, Taylor et al., 2016). Of note, such findings could result from parents having a higher threshold for recognising ADHD symptoms in daughters or clinicians having a higher threshold for diagnosing ADHD in females, and/or greater likelihood of diagnosing ADHD in females if their ADHD symptoms are accompanied by additional behavioural problems which make their ADHD symptoms more prominent (Martin et al., 2018).

Several methodological limitations exist in the study of sex differences in ADHD. First, many studies of ADHD are comprised of predominantly (or exclusively) male participants, limiting our understanding of ADHD in females; although efforts to investigate ADHD in females are increasing (e.g., Biederman et al., 1999, Hinshaw et al., 2006). Further, the DSM-IV criteria for ADHD are based primarily on observations of males (Lahey et al., 1994), and DSM-5 field studies included a greater percentage of males (Clarke et al., 2013). Second, much of our knowledge about sex effects in ADHD comes from clinical samples, yet referral bias related to sex suggests that studies in clinical samples may not provide the full picture regarding sex differences in ADHD, or generalise to the overall ADHD population. In addition, individuals whose ADHD is not diagnosed are absent from clinical samples and so findings may not be wholly applicable to females if they are more often ‘missed’. Moreover, previous meta-analyses highlight that clinic-referred girls may not be representative of non-referred girls in the same way that boys are (Gaub and Carlson, 1997, Gershon, 2002). Third, an additional obstacle in assessing sex differences in ADHD is that to date, nearly all studies of population or non-referred samples have relied on parent or teacher rating scales. If a male stereotype of ADHD is the norm, potentially only the most severe girls or those whose symptoms manifest as disruptive behaviours will be identified.

It is important, especially for clinical practice, to understand more about phenotypic differences in boys and girls with ADHD (Taylor et al., 2016), including sex differences beyond that of ADHD symptoms. One way to do this is to examine girls and boys from the general population with comparable numbers of ADHD symptoms and investigate which children meet diagnostic criteria to help understand whether different factors impact if boys and girls meet diagnostic threshold. One hypothesis is that if current diagnostic criteria and perceptions of ADHD characterise a male stereotype, and/or if there is a female protective effect, then girls may be less likely to meet diagnostic criteria or receive an ADHD diagnoses unless their symptoms are made more prominent by additional problems, for example greater emotional problems or school impairment.

To address the methodological issues detailed above, the current study examined data from a population-based twin sample, overcoming issues of referral and clinic bias. Separate trait and diagnostic measures of ADHD were available which extends methodology typically used in population-based samples. We examined what characteristics distinguish girls meeting diagnostic criteria (on the Parental Account of Childhood Symptoms [PACS]) from girls who do not despite high ADHD symptom levels on a rating-scale measure of ADHD, and if the same distinguishing characteristics operate in boys. We examined core ADHD symptom dimensions, co-occurring behavioural and emotional problems, and impairment. We also assessed sex-dependent biases in parental perceptions of ADHD symptoms by examining whether, despite their offspring meeting diagnostic criteria, parents systematically under- or over-rate relative to the PACS, and if this differs for boys and girls.

## 2 Method

### 2.1 Sample

**Fig. 1:**
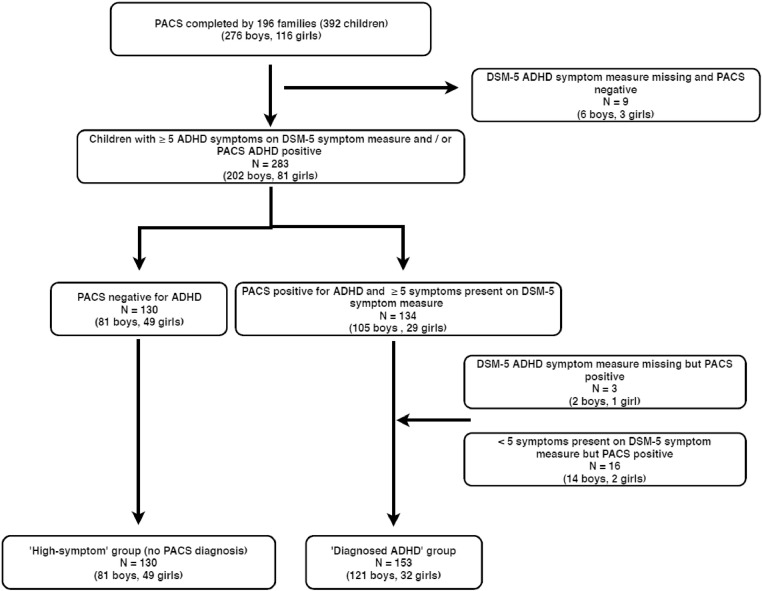
Flow diagram of how the ‘diagnosed ADHD’ and ‘high-symptom ADHD’ groups were derived for the current study.

Participants were 283 children: 153 (21% girls) who met DSM-5 research diagnostic criteria for ADHD based on the Parental Account of Childhood Symptoms (PACS) investigator-rated parental interview (*M* = 9.33 years, SD = 0.77) and 130 (38% girls) who showed a high level of ADHD symptoms based on parental report using DSM-5 symptom criteria but did not meet full diagnostic criteria (*M* = 9.52 years, *SD* = 0.88). We refer to the groups as the ‘diagnosed ADHD’ group and the ‘high-symptom’ group. Further details of the PACS is given in the measures section and details on how the diagnosis is made can be found in the supplementary material. We defined a high level of ADHD symptoms as the presence of 5 or more symptoms (out of 18), based on definitions used in previous studies (Biederman et al., 1996, Faraone et al., 2009, Faraone et al., 2006, Shankman et al., 2009). Of note, three children were missing their ADHD symptom score based on the DSM-5 rating-scale (2 boys, 1 girl) but met PACS diagnostic criteria, and 16 (14 boys, 2 girls) met PACS diagnostic criteria but had less than 5 symptoms present on the DSM-5 ADHD rating-scale; these children were included in the diagnosed group (see Fig. 1 for a flow chart of how the groups were derived).

Participants were part of the Developmental Pathways to Hyperactivity and Attention Deficit Study (PHAD), a sub-study of children at risk for ADHD identified from the Twins Early Development Study (TEDS), which is a population-based study of over 15,000 twin pairs born in the United Kingdom between 1994 and 1996, followed up prospectively from the age of 18 months. Details of TEDS is described elsewhere (Trouton et al., 2002), and details of the PHAD sample ascertainment can be found in the supplementary material. Briefly, children were screened for ADHD symptoms at age 7 years. Twins at risk were identified by at least one twin in each twin-pair scoring in the top 15% of the TEDS population for ADHD symptoms. 196 families with children identified as being at risk for ADHD (comprising 276 boys and 116 girls) completed the PACS ADHD diagnostic interview at the family home when the children were aged between 7–12 years (*M* = 9.42 years, *SD* = 0.84 years) (data was collected for both children in the twin pair regardless of whether one or both had been identified as being at risk for ADHD). Exclusion criteria were: autism spectrum disorder, learning disability, and neurological disability. The current study used a subsample of these children (*n* = 283) as detailed above.

### 2.2 Measures

#### 2.2.1 ADHD symptom measure

Parents completed a checklist assessing the 18 DSM-IV items for inattention (9 items) and hyperactivity/impulsivity (9 items) (American Psychiatric Association, 1994). Each item is rated on a four-point likert scale from 0 (not at all true) to 3 (very much true), with the highest possible score being 54. Of note, DSM-IV items for ADHD are the same in the most recent edition (DSM-5), and so we refer to DSM-5 for clarity.

#### 2.2.2 ADHD diagnostic measure

The Parental Account of Childhood Symptoms (PACS) was used to identify children who met diagnostic criteria for ADHD. The PACS is an investigator-rated semi-structured interview developed as a standardised measure for use in assessing and recording accurately the behaviours of children. Parents are asked to describe the behaviour of their child across a range of situations in relation to its frequency and severity, which is then scored according to a standardised operational scale (Chen and Taylor, 2006). As such, PACS can be considered a gold-standard tool for the research assessment of children with ADHD (Chen et al., 2008, Müller et al., 2011), and shows high inter-rater reliability (Chen et al., 2008). Details on the PACS diagnosis have been described elsewhere (Chen et al., 2008, Müller et al., 2011) and are included in the supplementary material.

#### 2.2.3 Co-occurring behavioural and emotional problems

The Strengths and Difficulties Questionnaire (SDQ) was used to assess behavioural and emotional problems using mothers ratings (Goodman, 1997). The SDQ comprises 5 subscales: emotional problems, conduct problems, hyperactivity, peer problems, and prosocial behaviour. The SDQ subscales can also be used to generate a ‘total problem’ score. The hyperactivity items were not used in this study as this behaviour is already measured.

#### 2.2.4 Impairment

The SDQ impact supplement comprises items related to overall distress and impairment which can be used to generate an impact score ranging from 0 to 10 (see supplementary material for further information). The PACS includes questions on both impairment and school behaviour, as related to the child's inattention and/or hyperactivity/impulsivity problems. The impairment items include: ‘problem is cause for concern’, ‘serious problem perceived/much concern’, ‘no control over behaviour’, ‘serious impairment/social impact of problem’, and ‘interviewer rates problem as markedly or severely abnormal’. Items were scored on likert scales which were used to create dichotomous variables for ‘yes’ or ‘no’ categories (see supplementary material for details of how these dichotomous variables were derived). We then calculated a total impairment score for each participant ranging from 0 to 5. School behaviour items were scored as ‘yes’ or ‘no’ and included: ‘child shows distress’, ‘problems getting on with others’, ‘difficulty concentrating’, ‘change of school due to problems’, ‘suspended or excluded’, ‘special educational provision’, ‘complaints about hyperactivity’, and ‘complaints about aggression’ (of note, this refers to physical aggression). We did not include the ‘difficulty concentrating’ item in analysis due to it being so closely linked with the definition of ADHD. ‘Change of school due to problems’ and ‘suspended or excluded’ were also omitted from analysis due to so few participants endorsing these items. A total school impairment score was derived for each participant ranging from 0 to 5.

### 2.3 Statistical analysis

We compared girls and boys meeting PACS DSM-5 diagnostic criteria to girls and boys with a high level of ADHD symptoms who did not meet diagnostic criteria on a-priori selected variables: parent-rated levels of inattention and hyperactivity/impulsivity, co-occurring behavioural and emotional problems, and impairment. We used linear regression models for continuous outcomes and logistic regression for binary outcomes. Age was controlled for in all analyses and we adjusted for familial clustering using the *cluster(variable)* function in Stata 14 (StataCorp, 2015). Effect sizes were established using Cohen's d for continuous variables (where: d≥0.20 is a small effect, d≥0.50 a medium effect, and d≥0.80 a large effect) and odds ratios for categorical variables.

To describe sex differences in the diagnosed and high-symptom groups, we compared girls versus boys meeting diagnostic criteria on the PACS, and high-symptom girls to high-symptom boys. To investigate which characteristics distinguish girls above and below the diagnostic threshold, and if the same distinguishing features operate in boys, we compared PACS diagnosed girls to high-symptom girls and PACS diagnosed boys to high-symptom boys by testing for an interaction between diagnostic group (diagnosed versus high-symptom) and sex (male versus female). A significant sex-by-diagnostic group interaction would indicate that certain characteristics are greater in diagnosed versus high-symptom individuals and in one sex compared to the other.

To elucidate potential sex biases in parent report of ADHD symptoms we examined whether, despite both sexes meeting diagnostic criteria, parents systematically under- or over-rate ADHD symptoms compared to the PACS interview assessment, and if this differs by sex. We tested for an interaction between sex and measure (parent-rating scale versus PACS) separately for total scores on the inattention and hyperactivity/impulsivity scale in the diagnosed group of children using linear regression. A significant interaction would suggest ADHD symptom score differs as a function of the type of measure and that this difference is greater in one sex compared to the other, or that the direction of the difference differs in girls and boys.

## 3 Results

Of the 153 children meeting PACS diagnostic criteria for ADHD, 121 were boys (79%) and 32 were girls (21%), giving a male-to-female ratio of 3.8:1. Of the 130 children in the high-symptom group, 81 were boys (62%) and 49 were girls (38%), a male-to-female ratio of 1.7:1. The ratio of diagnosed to high-symptom girls was 0.65:1 compared to 1.5:1 for boys. The diagnosed and high-symptom groups did not differ significantly in the total number of symptoms present based on the ADHD symptom measure (*p* = .10, *d* = 0.20), fulfilling a critical assumption on which the study design lies (i.e., that the diagnosed and high-symptom groups had comparable numbers of ADHD symptoms, therefore we can identify other factors that distinguish these groups.).

Among the children meeting PACS diagnostic criteria, girls had higher rated emotional problems (*p* = .04, *d* = 0.47) and were more prosocial (this difference showed a moderate effect size, but did not meet conventional levels of significance: *p* = .06, *d* = 0.42), compared with equivalent boys (Table 1). Girls meeting diagnostic criteria also had lower parent-rated impact scores (*p* = .03*, d* = −0.47), relating to overall distress and impairment, compared to boys meeting diagnostic criteria. Few characteristics distinguished girls and boys in the high-symptom group, except that girls were significantly more prosocial (*p* < .001, *d* = 0.78), had lower levels of conduct problems (*p* = .03, *d* = −0.45), and were less likely to have complaints about hyperactivity at school (*p* = .02, OR: 0.36 [95% CI: 0.15, 0.86]) compared to boys (Table 1).

**Table 1:** Characteristics of PACS diagnosed and high-symptom girls and boys. Mean (SD) unless otherwise stated.

	PACS diagnosed				High-symptom			
Characteristic^a^	Girls (n = 32)	Boys (n = 121)	*p*	Cohen's d	Girls (n = 49)	Boys (n = 81)	*p*	Cohen's d
**ADHD (parent-rated)**								
Inattention	17.62 (5.37)	16.47 (5.87)	.32	0.20	15.78 (4.58)	14.75 (4.94)	.25	0.22
Hyperactivity/ Impulsivity	15.39 (5.66)	15.37 (6.44)	.89	0.003	13.96 (5.63)	14.49 (4.85)	.65	−0.10
**Co-occurring difficulties**								
Emotional	**4.55 (2.75)**	**3.39 (2.37)**	**.04**	**0.47**	2.95 (2.10)	3.19 (2.47)	.63	−0.10
Conduct	3.24 (2.23)	3.72 (2.06)	.35	−0.23	**2.00 (1.69)**	**2.87 (2.13)**	**.03**	**−0.45**
Peer	3.34 (2.29)	3.58 (2.56)	.61	0.37	1.73 (1.87)	2.15 (2.18)	.33	−0.21
Prosocial	7.28 (2.39)	6.33 (2.22)	.06	0.42	**8.48 (1.97)**	**6.77 (2.40)**	**<.001**	**0.78**
Total Problem Score	11.14 (5.56)	10.69 (5.23)	.71	0.09	6.68 (3.61)	8.22 (5.08)	.11	−0.35
**Impairment**								
Total Impact Score (SDQ)	**1.79 (2.28)**	**2.93 (2.44)**	**.03**	**−0.47**	1.15 (1.46)	1.51 (2.18)	.31	−0.19
PACS total impairment	2.16 (1.69)	2.07 (1.84)	.83	0.05	0.73 (0.93)	0.89 (1.16)	.41	−0.15
**School Impairment (PACS)**								
Child shows distress (%)	43.8	49.6	.55	OR: 0.80 (0.38, 1.66)	42.9	29.6	.14	OR:1.78 (0.84, 3.78)
Problems getting on with others (%)	40.6	47.1	.52	OR: 0.78 (0.37, 1.66)	22.5	27.2	.53	OR:0.74 (0.29, 1.88)
Special educational provision (%)	40.6	42.2	.91	OR: 0.95 (0.40, 2.29)	16.3	12.4	.57	OR:1.38 (0.45, 4.26)
Complaints about hyperactivity (%)	59.4	58.7	.96	OR: 1.02 (0.47, 2.24)	**18.4**	**38.3**	**.02**	**OR:0.36 (0.15, 0.86)**
Complaints about aggression (%)	21.9	33.9	.72	OR: 0.54 (0.18, 1.62)	8.2	17.3	.16	OR:0.41 (0.12, 1.42)
Total school impairment	2.06 (1.37)	2.31 (1.46)	.37	−0.17	1.08 (1.17)	1.25 (1.33)	.44	−0.14

All models were adjusted for familial clustering and age.

Bold data signify statistical significance of the tests.

PACS = The Parental Account of Childhood Symptoms.

ADHD = Attention-deficit/hyperactivity disorder.

OR = Odds ratio (with 95% Confidence Intervals).

a Data were missing on some variables; all available data were used in analysis (see supplementary Table S1 for *n* available for each analysis).

### 3.1 Distinguishing characteristics of PACS diagnosed versus high-symptom girls, compared to boys

#### 3.1.1 PACS diagnosed versus high-symptom girls

Compared to high-symptom girls, girls meeting PACS diagnostic criteria had significantly higher reported levels of emotional (*p* = .02, *d* = 0.65), conduct (*p* = .04, *d* = 0.63), and peer problems (*p* = .002, *d* = 1.20), as well as higher total problem scores (*p* = .001, *d* = 0.95) (Table 2). The PACS diagnosed girls were also more impaired based on the PACS impairment measure (*p* < .001, *d* = 1.05) than the high-symptom girls, but the SDQ parent-rated impairment measure did not distinguish the two groups. At school, diagnosed girls received more complaints about hyperactive behaviour (*p* < .001, OR: 3.07 [95%CI: 2.42, 16.73]) and had higher overall school impairment scores (*p* = .001, *d* = 0.77). Girls meeting diagnostic criteria were also 3.89 times (95%CI: 1.19, 12.76) more likely to receive special educational provision (*p* = .03) than those with high ADHD symptom scores.

**Table 2:** Results of statistical analyses for within sex differences between diagnosed vs high symptom children, and sex-by-diagnostic status interaction analyses.

	PACS diagnosed vs high-symptom girls		PACS diagnosed vs high-symptom boys		Interaction (sex-by-diagnostic status)^b^	
Characteristic^a^	*p*	Cohen's d	*p*	Cohen's d	b/OR (95% CI)	*p*
**ADHD (parent-rated)**						
Inattention	.16	0.37	**.03**	**0.32**	0.10 (−2.77, 2.97)	.95
Hyperactivity/ Impulsivity	.45	0.25	.39	0.15	0.42 (−2.71, 3.54)	.79
**Co-occurring difficulties**						
Emotional	**.02**	**0.65**	.57	0.08	1.40 (−0.05, 2.86)	.06
Conduct	**.04**	**0.63**	**.01**	**0.41**	0.39 (−0.82, 1.61)	.53
Peer	**.002**	**1.20**	**<.001**	**0.60**	0.20 (−1.02, 1.41)	.75
Prosocial	.08	−0.55	.34	−0.19	−0.73 (−2.03, 0.56)	.27
Total Problem Score	**.001**	**0.95**	**.004**	**0.48**	1.99 (−0.96, 4.95)	.19
**Impairment**						
Total Impact Score (SDQ)	.20	0.33	**<.001**	**0.61**	−0.78 (−2.04, 0.47)	.22
PACS total impairment	**<.001**	**1.05**	**<.001**	**0.78**	0.24 (−0.59, 1.06)	.57
**School Impairment (PACS)**						
Child shows distress	.84	OR: 1.09 (0.46, 2.56)	**.004**	**OR: 2.38 (1.31, 4.30)**	0.45 (0.16, 1.25)	.12
Problems getting on with others	.05^c^	OR: 2.65 (0.98, 7.15)	**.003**	**OR: 2.72 (1.42, 5.21)**	1.04 (0.31, 3.44)	.95
Special educational provision	**.03**	**OR: 3.89 (1.19, 12.76)**	**<.001**	**OR: 5.23 (2.38, 11.50)**	0.69 (0.17, 2.74)	.60
Complaints about hyperactivity	**<.001**	**OR: 3.07 (2.42, 16.73)**	**.008**	**OR: 2.27 (1.24, 4.17)**	2.82 (0.91, 8.72)	.07
Complaints about aggression	.14	OR: 3.07 (0.70, 13.54)	**.02**	**OR: 2.58 (1.20, 5.55)**	1.31 (0.24, 6.99)	.76
Total school impairment	**.001**	**0.77**	**<.001**	**0.76**	−0.07 (−0.77, 0.63)	.85

All models were adjusted for familial clustering and age.

Bold data signify statistical significance of the tests.

PACS = The Parental Account of Childhood Symptoms.

ADHD = Attention-deficit/hyperactivity disorder.

OR = Odds ratio (with 95% Confidence Intervals).

a Data were missing on some variables; all available data were used in analysis (see supplementary Table S1 for *n* available for each analysis).

b Results for interaction analyses are presented as odds ratios (for School impairment measures, except total school impairment score) or unstandardised regression coefficients (for ADHD, co-occurring difficulties, and impairment) with 95% CIs.

c *p* = .054.

#### 3.1.2 PACS diagnosed versus high-symptom boys

Compared to the high-symptom boys, boys meeting PACS diagnostic criteria had greater parent-rated inattention (*p* = .03, *d* = 0.32) (Table 2), higher reported levels of conduct (*p* = .01, *d* = 0.41) and peer problems (*p* < .001, *d* = 0.60), and higher total problem scores (*p* = .004, *d* = 0.48). Greater impairment was demonstrated in boys meeting diagnostic criteria than high-symptom boys on all the impairment measures (see Table 2).

#### 3.1.3 Do the same distinguishing characteristics operate in girls and boys?

Overall there were no significant sex by diagnosis interactions, suggesting that many of the same factors distinguished high-symptom children from those who met diagnostic criteria in both boys and girls (Table 2). There were, however, some interactions nearing statistical significance in which certain characteristics played a larger role in distinguishing high-symptom girls from girls meeting diagnostic criteria, as compared to the equivalent groups of boys (Table 2, Supplementary Fig S1). Regarding co-occurring problems, girls who met PACS diagnostic criteria had more emotional problems compared with high-symptom girls (*d* = 0.65, *p* = .02), while this characteristic did not distinguish diagnosed and high-symptom boys (*d* = 0.08, *p* = .57; OR for interaction: 1.40 [95%CI: -0.05, 2.86], *p* = .058). Both girls and boys meeting PACS diagnostic criteria were distinguished from their high-symptom peers by greater total problem scores on the SDQ, and specifically conduct and peer problems, but the magnitude of the effect was greater in girls (total problems score: *d* = 0.95 vs 0.48; conduct problems: *d* *=* 0.63 vs 0.41; peer problems: *d* = 1.20 vs 0.60). In addition, magnitude of effect sizes suggested that prosocial behaviour distinguished diagnosed girls versus high-symptom girls but not boys, although this did not reach conventional levels of significance (girls: *d* = −0.55*, p* = .08 vs boys: *d* = −0.19*, p* = .34).

The SDQ total impact score (parent-rated measure of impairment) only distinguished between the PACS diagnosed and high-symptom boys, with almost double the effect size (boys: *d* = 0.61 vs girls: *d* = 0.33). Whereas the more objective PACS measure of impairment distinguished children meeting full diagnostic criteria from the children with high-symptoms who did not meet full diagnostic criteria in both girls and boys; however, the magnitude of this difference was greater in girls (*d* = 1.05 vs 0.78).

The total school impairment score distinguished PACS diagnosed from high-symptom children in both girls and boys, with similar effect sizes. PACS diagnosed boys were 2.38 times (95%CI: 1.31, 4.30) more likely to show distress at school than high-symptom boys, but PACS diagnosed and high-symptom girls were equally as likely to show distress at school (OR: 1.09, 95%CI: 0.46, 2.56). In both girls and boys, the likelihood of children meeting diagnostic criteria having complaints about their hyperactivity at school was greater than in high-symptom children, but the odds were greater for girls (OR: 3.07 [95%CI: 2.42, 16.73] vs 2.27 [1.24, 4.17]; OR for interaction: 2.82 [0.91, 8.72], *p* = .07). Problems getting on with others and complaints about aggression similarly distinguished diagnosed boys and girls from their high-symptom peers, but the difference was only significant for diagnosed versus high-symptom boys.

#### 3.1.4 Sensitivity analyses

To further ensure that the findings were not a function of differences in the number of ADHD symptoms between the groups being compared, analyses were re-run adjusted for this. This revealed the same pattern of results in terms of significance.

### 3.2 DSM-5 parent-rated ADHD symptoms compared to the PACS-reported symptoms in the diagnostic group

**Fig. 2:**
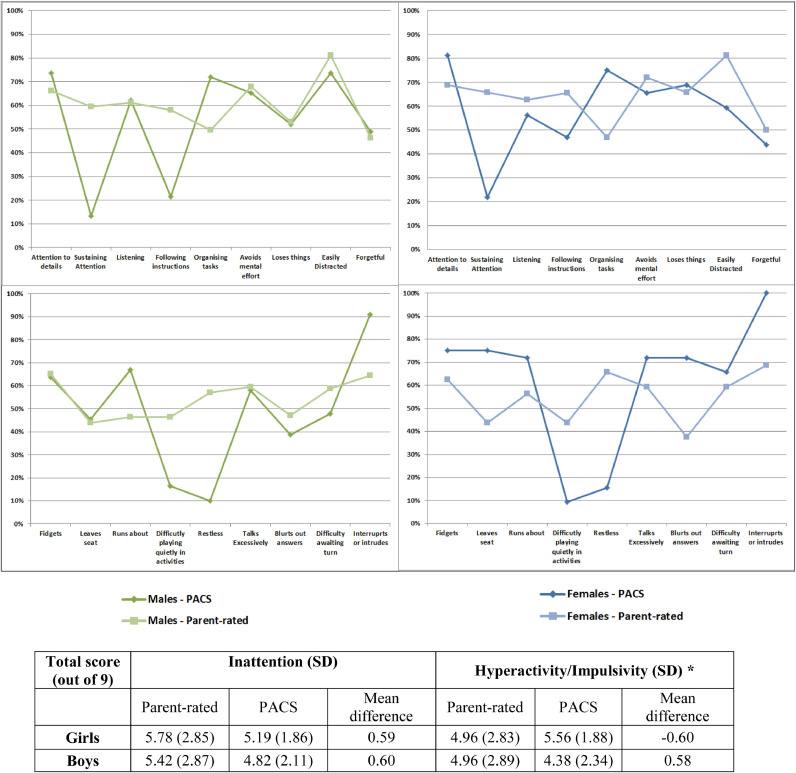
Frequencies of ADHD symptoms on the PACS interview compared to the parent-rating scale in children meeting full diagnostic criteria. *Significant sex-by-scale interaction (*p*<.02, 95% CI: -2.48 - -0.32).

A comparison of the frequency of parent rated DSM-5 ADHD symptoms compared to the same items on the PACS is illustrated in Fig. 2. In both boys and girls meeting diagnostic criteria, frequencies of inattentive symptoms were greater in the parent-rated scale compared to the PACS interview, apart from ‘attention to details’ (12.5% lower in the parent-rated scale in girls and 7.5% in boys), ‘organizing tasks’ (28.1% lower in girls and 22.3% in boys), ‘loses things’ in girls only (3.1% lower), and ‘listening’ and ‘forgetful’ in boys only (0.8% and 2.5% lower respectively).

Sex differences in the two measures were more noticeable for hyperactive/impulsive symptoms. In girls meeting diagnostic criteria, hyperactive/impulsive symptom frequencies were higher in the PACS compared to the parent-rated scale, except for ‘difficulty playing quietly in leisure activities’ (34.4% lower in the PACS) and ‘exhibits a consistent pattern of restlessness’ (50.1% lower). In diagnosed boys hyperactive/impulsive symptom frequencies were higher in the parent-rated scale than the PACS, except for leaves seat (1.7% lower on the parent-rated measure), runs about (20.6% lower), and interrupts or intrudes (26.5% lower). This pattern was reflected in the total score, and analyses found a significant sex-by-scale interaction for hyperactivity/impulsivity (*p*<.02, 95%CI: -2.48 - -0.32) indicating that parents tend to under-rate girls and over-rate boys for the presence of hyperactive/impulsive symptoms compared to the PACS (Fig. 2).

## 4 Discussion

In this study, we compared girls and boys who met full ADHD diagnostic criteria using an objective interview assessment to those who did not despite elevated levels of ADHD symptoms. When examining the factors that distinguished girls and boys who met full diagnostic criteria from their high-symptom peers, we found diagnosed girls had more additional problems than high-symptom girls, while this effect was less strong for boys. This could suggest girls with ADHD require a higher burden of other behavioural/emotional problems before they meet criteria for the disorder. We also found sex-dependent parental perceptions of ADHD behaviours and impairment.

Overall there were no significant sex by diagnosis interactions, suggesting that many of the same factors distinguished high-symptom children from those who met diagnostic criteria in both boys and girls. We found that girls meeting diagnostic criteria had higher rated emotional, conduct, and peer problems, total problem scores, and complaints about hyperactivity at school compared to the girls with high symptoms that did not pass the diagnostic threshold. Although similar differences were observed in boys (except for emotional problems) effect sizes were greater in girls, and were not due to the diagnosed and high-symptoms girls having a greater difference in ADHD symptoms compared to the equivalents groups of boys. The prominence of emotional symptoms in girls meeting diagnostic criteria suggests that this characteristic may be more important to the female phenotype and that girls may express their difficulties differently to boys. Higher rated emotional problems in girls than boys with ADHD has been shown previously (Novik et al., 2006). It is possible that emotional problems are not perceived to be as problematic compared to disruptive behaviours by individuals key in the diagnostic process, such as parents and teachers, reducing the likelihood of referral compared to children displaying disruptive behaviours. Further, perhaps emotional problems experienced by girls with ADHD are how they express or manifest their impairment, which could overshadow their ADHD symptoms in clinical assessment and lead to receiving alternative diagnoses more closely associated with the internal manifestation of symptoms (e.g., anxiety or depression), or delay time to diagnosis. Indeed, there is evidence to suggest that girls are diagnosed later (Agnew-Blais et al., 2016). This is problematic given the long-term outcomes associated with ADHD (Barkley, 2002, Shaw et al., 2012) and may be a particular issue if these symptoms result from the strain of compensating for their symptoms. It is important that the presence of emotional problems does not rule out an ADHD diagnoses (Quinn, 2008).

Parent-rated impairment using the SDQ did not distinguish between diagnosed versus high-symptom girls; yet it did in the equivalent groups of boys. This is an important finding with regard to sex differences in ADHD as referral based on parent concern requires recognition of impairment, yet parents appear to be less able to spot impairment among girls. One interpretation of these findings is that parents may not be as good at judging impairment in girls, highlighting that objective measures of impairment are especially important in the assessment of girls’ ADHD symptoms (Gaub and Carlson, 1997). Some diagnostic tools, such as rating-scale measures of ADHD or standardised parent interview assessments, may lead to underestimating girls’ impairment and contribute to their under-diagnosis, and parents may be less likely to take girls for assessment if they perceive them to be less impaired by symptoms compared to boys. Furthermore, this has implications for whether girls with ADHD receive appropriate treatments.

One characteristic that may influence the perception of impairment is prosocial behaviour. Not only is it clear that social functioning is likely to be linked with perceptions of impairment and coping, socially adaptive behaviour may mask symptoms and impairment to informants (Livingston and Happé, 2017). It appears that prosocial behaviour may have an influence on diagnostic status in girls but not boys. One interpretation of these findings it that in the presence of positive social behaviour, girls’ symptoms may be ‘masked’ making them appear less impaired, which could reduce the likelihood of girls with ADHD symptoms being referred and subsequently fewer girls compared to boys being diagnosed with ADHD. This hypothesis requires more research, along with the question of whether prosocial behaviour acts as a form of compensatory mechanism in girls with ADHD. It may also be that girls are more resilient to the impairments imposed by their ADHD symptoms and additional behavioural and emotional problems are therefore needed for impairment to be experienced.

Finally, we also found sex-dependent biases among parental perceptions of ADHD symptoms in children meeting diagnostic criteria. Parents under-rated diagnosed girls hyperactive/impulsive symptoms compared to the more objective accounts from the PACS, with the opposite pattern observed in boys. These findings suggest that sex-specific biases in perceptions of child behaviour may exist. As with the parental under-report of impairment in girls discussed previously, this also has clinical implications for the referral and subsequent diagnosis of girls with ADHD symptoms. Parents may be less likely to take girls for assessment if they perceive them to display less stereotypical ADHD behaviours (as well as being less impaired). It may also contribute to a systematic bias in diagnostic practice, as it may influence the identification and interpretation of symptoms by clinicians if they are relying on parental reports.

The present study has several strengths. We extended the methodology typically used in population-based studies by incorporating a comprehensive diagnostic interview and were therefore able to investigate sex differences in the factors that affect whether children with high ADHD symptoms meet diagnostic criteria in a population-based sample. In addition, the use of an objective, investigator-rated interview enabled investigation of potential sex-specific biases in parental report of ADHD symptoms. We have begun to tackle some of the possible contributing factors to the sex differences that could impact the referral and diagnostic process, but replication is needed.

However, some limitations should be noted. While sizeable for a study of its kind, there was a mismatch in the number of girls to boys, which is a common issue for studies of sex differences in ADHD. This reduces the power in interaction analyses to detect group differences and if these interactions were small they may have been missed; analyses should be repeated in a larger sample. This is also important to better elucidate findings that approached statistical significance. Also, in the current study we were not looking at who gets diagnosed in clinical settings, but rather investigating sex differences associated with meeting diagnostic criteria in a unique sample with less selection than studies of children who present to clinics. Unfortunately, as with many population-based studies, we do not have information on which individuals were actually referred, and so it is not possible to infer directly what the implications of the findings are on the referral and diagnostic process in clinical practice. Finally, it is important to note that our study was carried out in a twin sample. A concern when using twin samples is the generalisability of results to singletons. For example, relative to singletons, twins are more likely to have lower birth weight (Bhutta et al., 2002, Pettersson et al., 2016) and be born preterm, both of which show association with ADHD diagnosis (Aarnoudse-Moens et al., 2009, Bhutta et al., 2002, Johnson et al., 2010). Thus, it is important to replicate findings in non-twin samples.

It is clear that research is needed to identify the best screening methods, most accurate informants, and most appropriate thresholds for the diagnosis of ADHD, which may differ for boys and girls (Rucklidge, 2010). It is likely that the same instruments should be used for boys and girls, but with symptom examples that describe how symptoms may be expressed differently in boys and girls (e.g., girls may display their hyperactivity as inappropriate excessive talking and giggling Ohan and Johnston, 2005, Quinn and Wigal, 2004) and possibly with the addition of items that are more sensitive to the manifestation of ADHD in girls (Arnold, 1996). For example, items that relate better to social functioning and emotionality, and that are better placed to assess more internalising symptoms (Skogli et al., 2013). Perhaps this is timely given the ADHD diagnostic criteria are primarily based on studies in males (Clarke et al., 2013, Lahey et al., 1994), and that it has been shown that parents perceive the ADHD symptom items as being descriptive of boys (Ohan and Johnston, 2005).

In summary, these data suggest that factors which distinguish girls who meet full ADHD diagnostic criteria from high-symptom peers who do not may be somewhat sex specific, with additional behavioural and emotional problems playing a larger role in distinguishing diagnosed from high-symptom girls than the equivalent male comparison. Additionally, we found different parental perceptions of ADHD behaviours as shown by our comparison of parent report to a more objective measure of ADHD symptoms. Such differences may explain why girls are less likely to be referred for their ADHD behaviours. This may also contribute to the relatively low recognition rate of ADHD in girls in clinical practice if girls with ADHD are perceived to display less stereotypical disruptive ADHD behaviours and perceived as less impaired by symptoms than boys, especially in the presence of socially adaptive behaviour and more internalising emotional symptoms. From a clinical perspective, our findings highlight the importance of detailed interview assessments in the diagnostic process, especially for girls who may not be identified with rating-scale measures which are more subject to sex biased perceptions of behaviour, and that emotional problems should not be used to rule out an ADHD diagnoses.

## Appendix Supplementary materials

Image, application 1

Image, application 2
