# Supplementary material for: Do different factors influence whether girls versus boys meet ADHD diagnostic criteria? Sex differences among children with high ADHD symptoms
Source: Psychiatry Res. 2019 Feb;272:765–73. doi: 10.1016/j.psychres.2018.12.128 (PMC6401208; doi:10.1016/j.psychres.2018.12.128)
Supplement: Supplementary file 2 [file mmc2.zip › mmc2.pdf]

Online Supplementary Material

Mowlem, F.D., Agnew-Blais, J., Taylor, E., & Asherson, P. Do different factors influence whether girls versus boys meet ADHD diagnostic criteria? Sex differences among children with high ADHD symptoms. *Psychiatry Research.*

Additional sample information

The Developmental Pathways to Hyperactivity and Attention Deficit Study (PHAD)

PHAD is a spin-off study from the larger Twins Early Development Study (TEDS) [1]. The primary aim of PHAD was identifying early neuroimaging and cognitive markers underlying risk for ADHD. Twins from the TEDS sampling frame were screened for ADHD symptoms at age 7 years using combined parent and teaching ratings on the hyperactivity/inattention subscale of the Strengths and Difficulties Questionnaire (SDQ), plus three additional items addressing attention and hyperactivity and impulsivity problems ('notices small details', 'has difficulty completing activities', 'has difficulty waiting for things'). The SDQ is frequently used in both clinical and research assessments of ADHD and as a measure to detect children at high risk of mental health problems especially using multiple informants [2–4]. Twins at risk of ADHD were identified if at least one twin in each twin-pair scored in the top 15% of the TEDS population. Families were excluded if they had withdrawn from TEDS or were uncontactable, were involved in other TEDS spin-off studies, or if medical exclusions applied. Opposite sex-pairs were excluded as the original objective of the study was to compare within twin-pair cognitive and neuroimaging findings for ADHD, while removing the potential confounding effect of sex differences. This led to 861 families being selected from TEDS where at least one twin was at risk of ADHD. Of these, 690 families agreed to participate in a further screening telephone interview, based on which 200 families were excluded: 67 due to reports of at least one twin having a learning disability, autistic spectrum disorder, or a neurological disability, and 133 who reported having no problems at home or school, or problems in only one setting. Of the remaining 490 families, 345 parents completed an ADHD symptom checklist based on DSM-IV criteria [5], based on which 138 families were excluded where neither twin met the required symptom threshold (score >22). This left a sample of 207 families with at least one twin with high levels of ADHD symptoms. 196 families with children identified as being at risk for ADHD (comprising 276 boys and 116 girls) completed the Parental Account of Childhood Symptoms (PACS) diagnostic interview.

Additional information on study measures

PACS diagnosis

As in previous studies using the PACS [6, 7], an ADHD diagnostic algorithm combined data from the PACS and the Conners' teacher-rating scale for DSM-IV ADHD symptoms to apply a research diagnosis of ADHD. Children were 'diagnosed' if sufficient items were identified to fulfil DSM-5 criteria (6 or more symptoms from at least one symptom domain), and both impairment (based on severity of symptoms identified in the PACS) and pervasiveness (based on the presence of ADHD symptoms in more than one setting using information from the PACS and the Teacher Conners') were present. Situational pervasiveness outside the home setting is also captured in the PACS interview.

Impairment: SDQ Impact supplement

Parents are first asked 'Overall, do you think that your child has difficulties in one or more of the following areas: emotions, concentration, behaviour or being able to get on with other people?'. Subsequent items include: 'Difficulties upset or distress child', 'Interfere with home life', 'Interfere with friendships', 'Interfere with classroom learning', and 'Interfere with leisure activities'. If 'no' is answered to the first question then the subsequent questions are not asked and the impact score is automatically scored as '0'.

Impairment: PACS impairment

PACS impairment items are scored on likert scales which were used to create dichotomous variables for 'yes' or 'no' categories. Below are details of how the dichotomous variables were derived from the likert scale corresponding to each item:

Item	Likert scale scoring	Dichotomous variable
Problem is cause for concern	0 = no cause for concern<br>1 = cause for concern<br>8 = not applicable – no problem behaviour	0, 8 = no<br>1 = yes
Serious problem perceived/much concern	0 = no problem perceived/no concern<br>1 = minor problem perceived/slight concern<br>2 = serious problem perceived/much concern	0, 1= no<br>2 = yes
No control over behaviour	0 = can almost always control behaviour<br>1 = sometimes has control over behaviour<br>2 = no control over behaviour<br>8 = not applicable	0, 1, 8 = no<br>2 = yes
Serious impairment/social impact of problem	0 = no impairment<br>1 = minor impairment<br>2 = serious impairment<br>8 = not applicable	0, 1, 8 = no<br>2 = yes
Interviewer rates problem as markedly or severely abnormal	0 = normal<br>1 = mildly abnormal<br>2= markedly abnormal<br>3 = severely abnormal<br>8 = not applicable	0, 1, 8 = no<br>2, 3 = yes

Supplementary Table S1 Available *n* for analyses as a function of group and variable (characteristic)

	PACS diagnosed		High-symptom	
**Characteristic[a]**	**Girls (n=32)**	**Boys (n=121)**	**Girls (n=49)**	**Boys (n=81)**
**ADHD (parent-rated)**				
Inattention	31	119	49	81
Hyperactivity/ Impulsivity	31	119	49	81
**Co-occurring difficulties**				
Emotional	29	110	40	78
Conduct	29	110	40	78
Peer	29	110	40	78
Prosocial	29	110	40	78
Total Problem Score	29	110	40	78
**Impairment**				
Total Impact Score (SDQ)	28	107	40	74
PACS total impairment	32	121	49	81
**School Impairment (PACS)**				
Child shows distress (%)	32	121	49	81
Problems getting on with others (%)	32	121	49	81
Special educational provision (%)	32	121	49	81
Complaints about hyperactivity (%)	32	121	49	81
Complaints about aggression (%)	32	121	49	81
Total school impairment	32	121	49	81

Supplementary Fig. S1 Graphical representation of the sex-by-diagnostic status interactions that approached significance

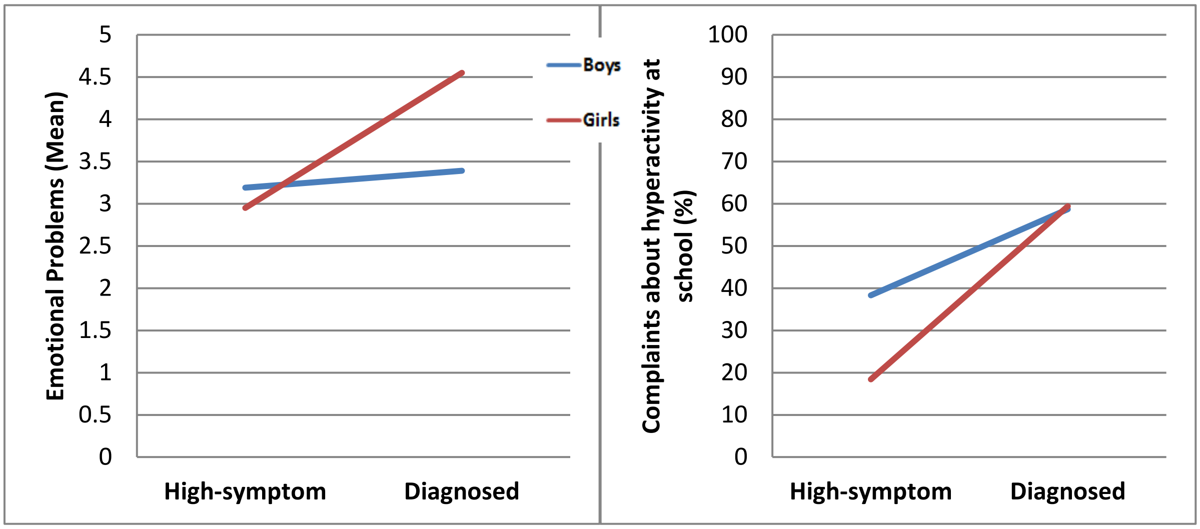

1. Trouton A, Spinath FM, Plomin R (2002) Twins' Early Development Study (TEDS): a multivariate, longitudinal genetic investigation of language, cognition and behavior problems inchildhood. Twin Res 5:444–448 . doi: 10.1375/twin.5.5.444
2. Goodman R, Ford T, Simmons H, et al (2000) Using the Strengths and Difficulties Questionnaire (SDQ) to screen for child psychiatric disorders in a community sample. Br J Psychiatry 177:534–539 . doi: 10.1192/bjp.177.6.534
3. Carballo JJ, Rodríguez-Blanco L, García-Nieto R, Baca-García E (2014) Screening for the ADHD Phenotype Using the Strengths and Difficulties Questionnaire in a Clinical Sample of Newly Referred Children and Adolescents. J Atten Disord. doi: 10.1177/1087054714561858
4. Huss M, Hölling H, Kurth BM, Schlack R (2008) How often are german children and adolescents diagnosed with ADHD? Prevalence based on the judgment of health care professionals: Results of the german health and examination survey (KiGGS). Eur Child Adolesc Psychiatry 17:52–58 . doi: 10.1007/s00787-008-1006-z
5. American Psychiatric Association (1994) Diagnostic and statistical manual of mental disorders (DSM-IV), 4th ed. American Psychiatric Association, Washington, DC
6. Chen W, Zhou K, Sham P, et al (2008) DSM-IV combined type ADHD shows familial association with sibling trait scores: a sampling strategy for QTL linkage. Am J Med Genet Part B, Neuropsychiatr Genet 147B:1450–1460 . doi: 10.1002/ajmg.b.30672
7. Müller UC, Asherson P, Banaschewski T, et al (2011) The impact of study design and diagnostic approach in a large multi-centre ADHD study. Part 1: ADHD symptom patterns. BMC Psychiatry 11:54
